# Cellulitis of the Neck

**Published:** 1879-12

**Authors:** 


CELLULITUS OF THE NECK.—A consideration of the literature of this subject, together with results of his own experience, lead Mr. R. W. Parker to suggest the following as a suitable classification of this affection: 1. Idiopathic (angina Ludovici); 2. Traumatic, occurring after injuries and operations; 3. Extension of contiguous inflammation. The idiopathic form is a dangerous, and, most frequently, a fatal disease, occurring concurrently with other and more common form of epidemic disease, such as erysipelas, measles, scarlet fever and "putrid sore throat," yet, in the most marked cases, the tonsils and pharynx are described as having been normal. The treatment should be bold and prompt; free incisions into the indurated cellular tissue, deep enough to open up the various sheaths of the deep cervical fascia, must be carefully made, so as to let out the sanious ichor, and thus prevent its burrowing. Surgeons are well acquainted with the traumatic form of cervical cellulitis which is apt to make its appearance after all kinds of injuries to the neck. In its treatment Mr. Parker has found the greatest benefit from the use of liquor plumbi made into a lotion with milk instead of distilled water. The most common form of the disease is that due to the extension of a contiguous inflammation. In its treatment, the fact must be borne in mind that much of the danger arises from the inflammation being seated beneath the deep cervical fascia, the undermost layer of which is continuous with the pericardium.—*The Lancet.*

